# Crystallographic analysis and phasing of iron-assimilating protein 1 (FEA1) from *Chlamydomonas reinhardtii*

**DOI:** 10.1107/S2053230X21003952

**Published:** 2021-04-28

**Authors:** Linda Juniar, Vida Adlfar, Michael Hippler, Hideaki Tanaka, Genji Kurisu

**Affiliations:** a Institute for Protein Research, Osaka University, Yamada-oka 3-2, Suita, Osaka 565-0871, Japan; b Institute of Plant Biology and Biotechnology, University of Münster, 48143 Münster, Germany

**Keywords:** iron transport, FEA1, SAD, *Chlamydomonas reinhardtii*

## Abstract

As an essential component of protein cofactors, iron is important for all photosynthetic organisms. In *Chlamydomonas reinhardtii*, iron levels are strictly controlled by proteins such as iron-assimilating protein 1 (FEA1). This periplasmic protein is expressed under conditions of iron deficiency, but its mechanisms of function remain unknown. Because FEA1 has no amino-acid similarity to protein structures in the Protein Data Bank, its crystal structure cannot be solved by molecular replacement. Here, recombinant FEA1 protein lacking the N-terminal signal sequence was successfully purified and crystals of apo FEA1 were obtained by hanging-drop vapor diffusion. Neither selenomethionine substitution nor heavy-atom derivatization was successful; therefore, the phase problem of FEA1 crystals was solved by the native sulfur SAD method using long-wavelength X-rays (2.7 Å). Laser-cutting technology was used to increase the signal-to-noise ratio and derive an initial structure. This study will lead to further structural studies of FEA1 to understand its function and its links to the iron-assimilation pathway.

## Abstract 2

In *Chlamydomonas reinhardtii*, iron levels are strictly controlled by iron-assimilating protein 1 (FEA1). Recombinant FEA1 protein was successfully purified and crystallized by hanging-drop vapor diffusion. The phase problem was solved by the native sulfur SAD method using long-wavelength X-rays (2.7 Å). Laser-cutting technology was used to increase the signal-to-noise ratio and derive an initial structure.

## 1. Introduction   

The green alga *Chlamydomonas reinhardtii* is important in many fields of research, including metal metabolism. Metals play essential roles in cells as part of protein cofactors; therefore, their concentrations are tightly controlled as an excess amount can be toxic, while a deficiency leads to inactive metalloenzymes (Hanikenne, 2003 ▸; Merchant *et al.*, 2006 ▸). As a photosynthetic organism, *C. reinhardtii* has various iron-dependent enzymes with vital functions in electron pathways, reactive oxygen detoxification, fatty-acid metabolism and amino-acid biosynthesis (Glaesener *et al.*, 2013 ▸).

Rubinelli and coworkers initially characterized the regulation of the expression of HCR1-like protein (H43) (now called iron-assimilation protein 1; FEA1), which is induced by an iron-deficient medium (Rubinelli *et al.*, 2002 ▸). FEA1 is a homologue of HCR1 from the marine alga *Chlorococcum littorale*. Both proteins are highly induced by high CO_2_ levels and iron deficiency (Rubinelli *et al.*, 2002 ▸; Kobayashi *et al.*, 1997 ▸; Baba *et al.*, 2011 ▸; Sasaki *et al.*, 1998 ▸). Studies have shown that the abundance of FEA1 mRNA and protein is greatly increased under conditions of iron deficiency in *C. reinhardtii* (Allen *et al.*, 2007 ▸; Urzica *et al.*, 2012 ▸). Importantly, it has been shown that the FEA1 protein is the major protein secreted into the periplasm by iron-deficient *C. reinhardtii* cells, and is expressed coordinately with the *FRE1* and *FOX1* genes (Allen *et al.*, 2007 ▸). It has also been shown to be N-glycosylated (Mathieu-Rivet *et al.*, 2013 ▸).

The exact molecular mechanism of FEA1 in iron assimilation by *C. reinhardtii* is unknown. Its currently proposed function is to concentrate iron in the periplasmic space in the vicinity of iron transporters in order to increase the efficiency of iron uptake under conditions of iron deficiency (Allen *et al.*, 2007 ▸). Although FEA1 is not homologous to classical transferrins, a recent study identified ISIP2A as a phyto-transferrin in diatoms functioning in carbonate-dependent iron assimilation, and phylogenic analysis indicates that FEA1 and ISIP2A are related (McQuaid *et al.*, 2018 ▸), potentially offering new hypotheses about the function of *C. reinhardtii* FEA1.

Structural characterization of FEA1 will be important to gain a deeper understanding of its role in the iron-assimilation pathway. In crystallography, protein structures are mostly solved by molecular replacement; however, proteins with no homology to structures in the Protein Data Bank (PDB) require experimental phasing. One such phasing method is single-wavelength anomalous dispersion (SAD) using selenomethionine (SeMet) or heavy-atom derivatized crystals. However, if derivatization with SeMet or additional heavy metals, such as mercury or platinum, is not possible, the only option left is native SAD using the S atoms of methionine or cysteine (S-SAD), a method that has been under development since its first reported use to determine the structure of crambin in 1981 (Hendrickson & Teeter, 1981 ▸). In fact, a number of protein structures deposited in the PDB have been solved by native SAD (Rose *et al.*, 2015 ▸). In SAD, using a long wavelength (2.7 Å) can increase the anomalous signal (*f*′′ of sulfur is 1.5 e^–^) relative to shorter wavelengths; however, both the crystal thickness and the solvent surrounding the crystal may cause strong absorption effects prior to data collection at long wavelengths (Basu *et al.*, 2019 ▸). Laser-cutting technology might be the answer to this problem: it can improve the data quality in crystallographic data processing, decrease the dispersion of angle-dependent scale factors and increase the signal-to-noise ratio (Kitano *et al.*, 2005 ▸; Basu *et al.*, 2019 ▸).

We found that FEA1 can only be crystallized in the apo form without bound Fe atoms; therefore, we first attempted experimental phasing using SeMet and heavy-atom derivatization. As these approaches failed, we then used S-SAD to phase the diffraction data from FEA1 crystals. Here, we report the crystallization and successful phasing of FEA1 using S-SAD at a long wavelength of 2.7 Å coupled with laser-cutting technology.

## 2. Materials and methods   

### 2.1. Macromolecule production   

The gene fragment encoding the FEA1 protein (Gln19–Ala362 of UniProt Q9LD42) was inserted into plasmid pASK-IBA2 (IBA). The FEA1 plasmid was designed to express the periplasmic protein with a Strep-tag at the C-terminus. Information on plasmid construction and expression is summarized in Table 1 ▸. The recombinant plasmid pASK-IBA2 for FEA1 was transformed into *Escherichia coli* Rosetta 2 (DE3) cells. A preculture was prepared from a fresh colony incubated at 26°C overnight and was used to inoculate the main culture. The cells were grown in Luria–Bertani medium at 26°C and, when the optical density at 550 nm (OD_550_) had reached 0.5–0.6, protein expression was induced by adding anhydrotetracycline (to a final concentration of 200 ng ml^−1^) and incubating at 26°C for 16–18 h. The cell pellet was collected by centrifugation at 4000*g* at 4°C for 12 min.

For protein purification, the cells were suspended in 100 m*M* Tris–HCl pH 8.0, 500 m*M* sucrose, 2 mg ml^−1^ polymyxin B sulfate and incubated on ice for 30 min. The periplasmic protein was collected by centrifugation at 149 000*g* at 4°C for 30 min. The supernatant was applied onto an equilibrated Strep-Tactin column (IBA), contaminants were washed through with 100 m*M* Tris–HCl pH 8.0, and the FEA1 protein was eluted with 100 m*M* Tris–HCl pH 7.5, 2.5 m*M* desthiobiotin. For further purification, the eluted fraction was applied onto an SP HP column (GE Healthcare) and the protein was eluted by a 0–500 m*M* linear gradient of NaCl in 100 m*M* Tris–HCl pH 7.5. The purified FEA1 protein was concentrated in 40 m*M* Tris–HCl pH 7.5 using an Amicon Ultra-15 Centrifugal Filter Unit (10 000 molecular-mass cutoff; Merck Millipore). The protein concentration was estimated by the bicinchoninic acid (BCA) assay.

### 2.2. Crystallization   

Initial screening for FEA1 crystals was performed by the sitting-drop vapor-diffusion method using ten commercial screening kits, Crystal Screen, Crystal Screen 2, PEG Rx 1 and 2, PEG/Ion and PEG/Ion 2 (Hampton Research, USA) and Wizard I, II, III and IV (Rigaku, USA), in a 96-well plate using a Mosquito LCP crystallization robot (TTP Labtech). The FEA1 concentration used for crystallization was 10 mg ml^−1^ in 40 m*M* Tris–HCl pH 7.5. Droplets consisting of 0.2 µl protein solution and 0.2 µl reservoir solution were equilibrated against 80 µl reservoir solution at 4 and 20°C. Based on the crystals obtained in the initial screening, crystallization was optimized by the hanging-drop vapor-diffusion method using different precipitating agents and additives around the initial conditions. Ultimately, a mixture of 1 µl protein sample and 1 µl reservoir solution was equilibrated against 150 µl reservoir solution, and crystals of FEA1 were obtained in 200 m*M* ammonium sulfate, 100 m*M* imidazole–HCl pH 6.5, 11%(*w*/*v*) PEG 3350, 30%(*v*/*v*) MPD at 4°C. The crystallization conditions are summarized in Table 2 ▸.

### 2.3. Data collection and processing   

An FEA1 crystal was picked up by a loop and quickly cooled in liquid nitrogen for data collection. Diffraction data were collected on beamline BL44XU at SPring-8, Harima, Japan using an EIGER X 16M system (Dectris, Baden, Switzerland). S-SAD data were collected on beamline BL-1A at the Photon Factory (PF), Tsukuba, Japan using an EIGER X 4M system (Dectris, Baden, Switzerland). To increase the signal-to-noise ratio, the loop was cut by a laser on station AR-NW12A at the PF. All diffraction images were collected at 100 K and were processed, merged and scaled using *XDS*/*XSCALE* (Kabsch, 2010 ▸). The initial model was determined by SAD using *autoSHARP* followed by *autoBUSTER* (Vonrhein *et al.*, 2007 ▸; Bricogne *et al.*, 2011 ▸). It was then used as a search model to solve a native data set collected at a higher resolution on beamline BL44XU at SPring-8, Hyogo, Japan by molecular replacement using *Phaser* (McCoy, 2017) as part of the *CCP*4 suite (Winn *et al.*, 2011 ▸). Data-collection and processing statistics are summarized in Table 3 ▸.

## 3. Results and discussion   

**Figure 1:**
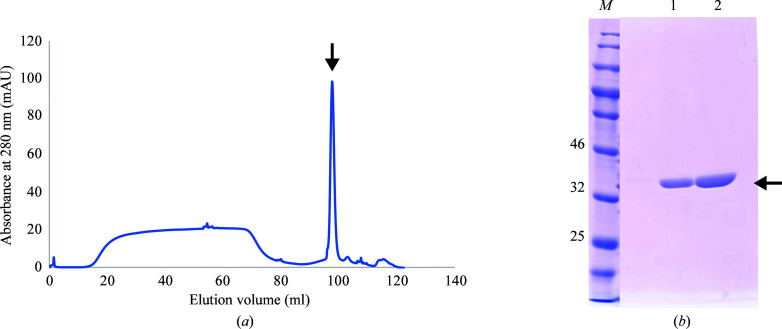
Purification of FEA1 by cation-exchange chromatography. (*a*) Chromatogram and (*b*) SDS–PAGE analysis of purified FEA1. Lane *M*, protein markers (labeled in kDa); lanes 1–2, purified FEA1.

FEA1 is a periplasmic protein encoded by the *h43* gene (UniProt Q9LD42) in *Chlamydomonas*. Notably, the construct used in this study comprised amino-acid residues Gly19–Ala362 of FEA1 because the first 18 residues of the N-terminal region are considered to be the signal peptide and were thus omitted. FEA1 was constructed in a pASK-IBA2 vector and heterologously expressed in *E. coli* Rosetta (DE3) cells as a periplasmic protein with a Strep-tag (LEVDLQGDHGLSAWSHPQFEK) attached to the C-terminus. The recombinant protein was purified using a Strep-Tactin column, followed by cation-exchange chromatography using an SP HP column. The purified protein was checked by 12.5% SDS–PAGE, which showed a single band with a molecular weight of 38.6 kDa (Fig. 1 ▸).

**Figure 2:**
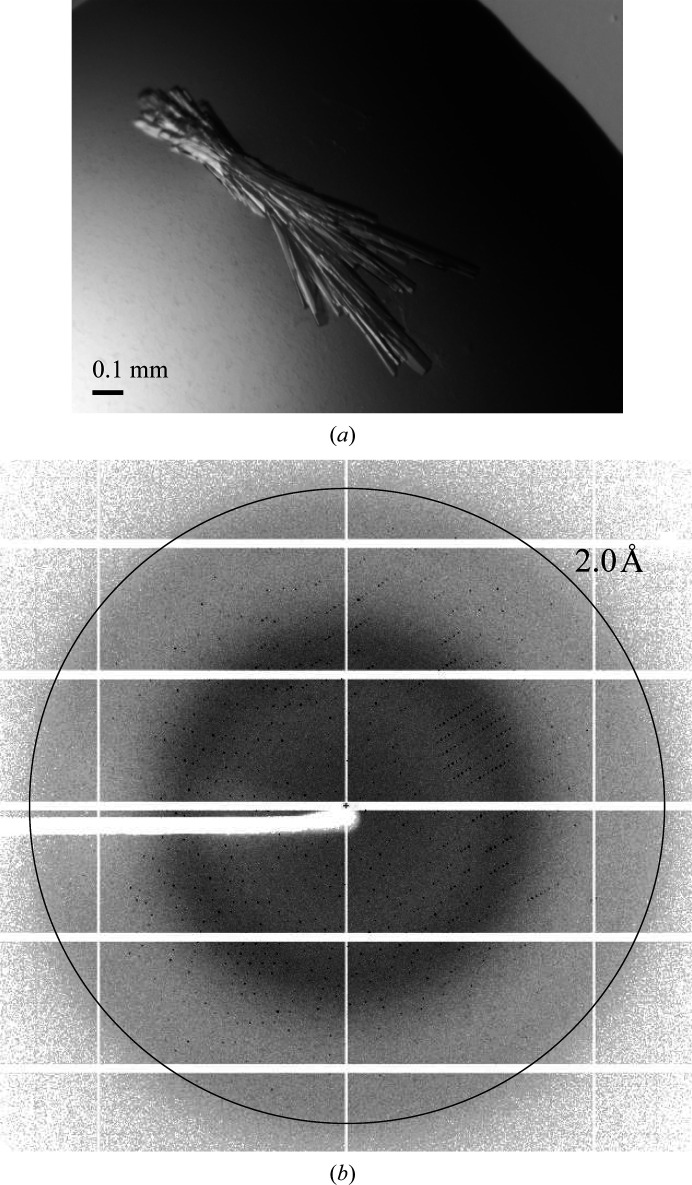
(*a*) A crystal of FEA1. (*b*) Diffraction image of an FEA1 crystal recorded on beamline BL44XU at SPring-8, Hyogo, Japan.

The purified protein was crystallized by the hanging-drop vapor-diffusion method in 200 m*M* ammonium sulfate, 100 m*M* imidazole–HCl pH 6.5, 11%(*w*/*v*) PEG 3350, 30%(*w*/*v*) MPD at 4°C. The crystals were obtained with good reproducibility and diffracted well to 1.9 Å resolution, as shown in Fig. 2 ▸. Because there are no proteins in the PDB with a similar amino-acid sequence, we carried out experimental phasing. Firstly, we tried to express SeMet-substituted FEA1 under conditions of methionine-pathway inhibition. However, the expression level was very low, and the amount of sample obtained was not sufficient for crystallization. Next, several heavy-atom derivatives were prepared, including those with thallous acetate, mercury(II) acetate, potassium tetrachloro­platinate(II), iron(III) chloride and iron(II) sulfate. Unfortunately, none of them were successful in solving the phase problem.

**Figure 3:**
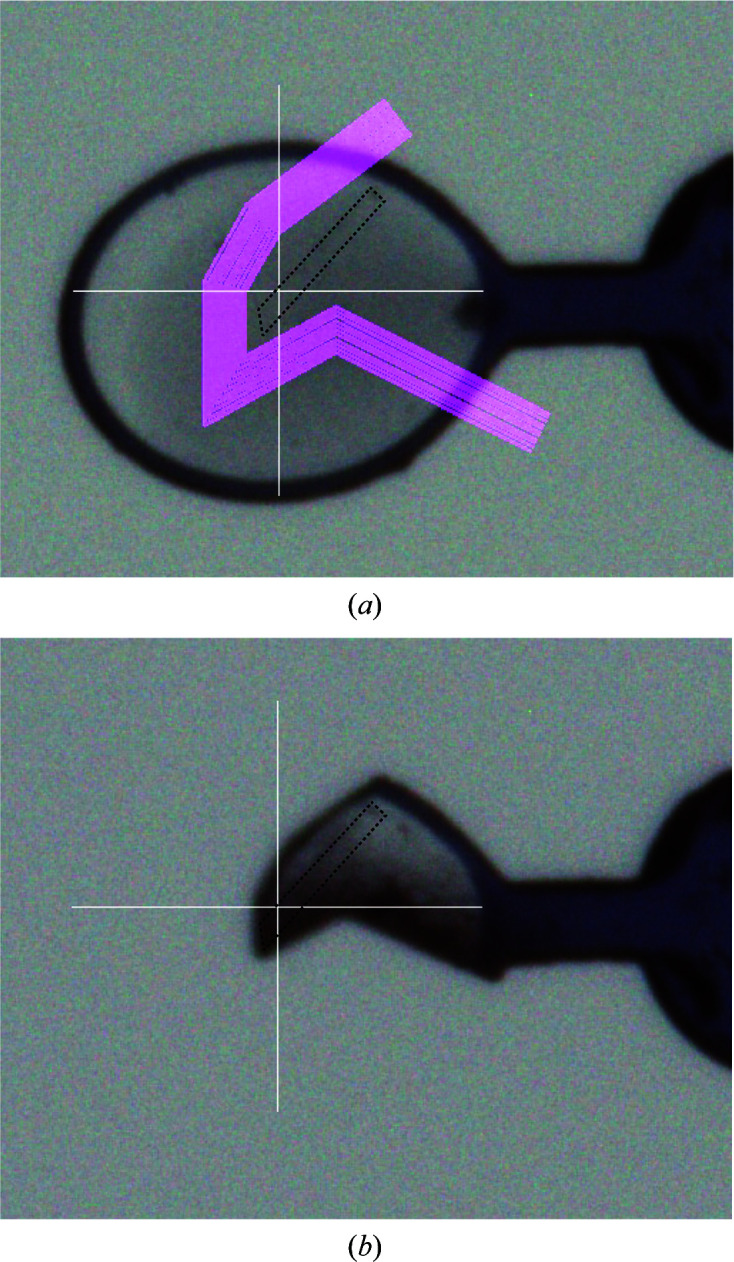
Laser cutting to decrease the solvent content of the loop. (*a*) Original loop before laser cutting and the cutting-line pattern (pink line); (*b*) the same loop after laser cutting. The shape of the FEA1 crystal is shown by a black dotted line.

**Figure 4:**
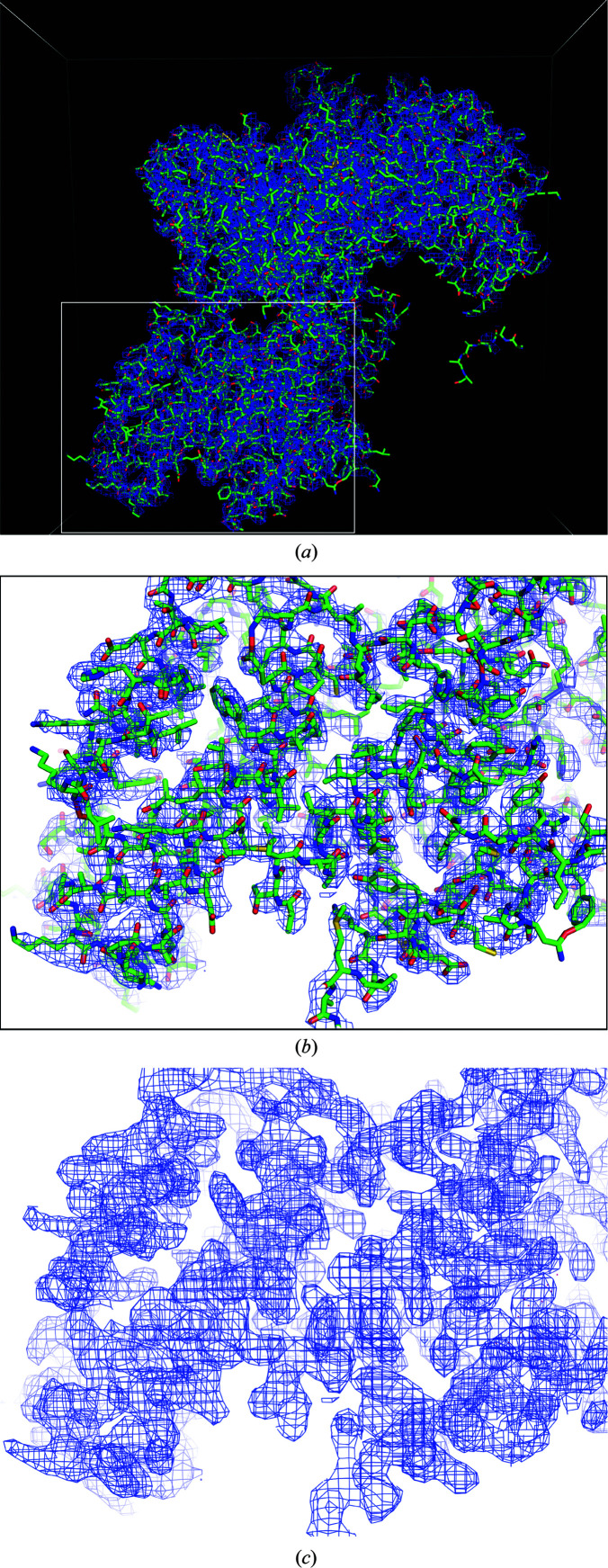
Initial structure of FEA1 solved by S-SAD. (*a*, *b*) The electron-density map is contoured around the initial structure. (*c*) The experimental electron-density map from *autoBUSTER*. The figures were generated using *PyMOL.*

Because FEA1 has 13 S atoms, we next tried S-SAD to determine the initial phase. Diffraction data for S-SAD were collected using an EIGER X 4M system (Dectris, Baden, Switzerland) at the long wavelength of 2.7 Å on beamline BL-1A at the Photon Factory, Tsukuba, Japan. The diffraction data were processed using *XDS*. 27 unmerged data sets were then analyzed using *XSCALE_CLUSTER* to find isomorphous data sets. In the first attempt, eight data sets were used from the largest isomorphous cluster from one crystal. The data sets comprised 3600 frames with oscillation angles of 0–360° taken at different crystal positions and kappa angles; however, a native SAD solution was not obtained. In the second attempt, a loop was cut by a laser under the cryogenic conditions to remove the solvent on station AR-NW12A at the Photon Factory, Tsukuba, Japan prior to data collection in order to reduce scattering by the solvent. The crystal of FEA1 was a thin rod-shaped crystal with dimensions of 0.2 × 0.024 mm as shown by a black dotted line in Fig. 3 ▸. The solvent around the crystal was removed as much as possible, and the final distance between the cutting line and the crystal was about 0.032–0.039 mm. After the laser-cutting process, 29 data sets were collected using the solvent-removed crystals. 16 isomorphic data sets from the first and second data collections were then merged and scaled at 2.6 Å resolution using *XSCALE*. Finally, the phase was solved by SAD using *autoSHARP* followed by *autoBUSTER* (Vonrhein *et al.*, 2007 ▸; Bricogne *et al.*, 2011 ▸). There were 1060 residues in seven chains, with 1032 residues of the FEA1 sequence in the initial structure (Fig. 4 ▸).

The crystal of FEA1, which diffracted to 1.9 Å resolution, belonged to space group *C*2, with unit-cell parameters *a* = 85.75, *b* = 155.94, *c* = 129.53 Å, β = 102.27°. The Matthews coefficient of the FEA1 crystal was 3.37 Å^3^ Da^−1^, with three molecules in the crystallographic asymmetric unit and 63.5% solvent content (Matthews, 1968 ▸). The initial structure from the SAD solution was used as a search model for higher resolution data sets using *Phaser* (McCoy, 2017 ▸). Model rebuilding and refinement are ongoing.

In summary, we have used S-SAD and laser-cutting technology to solve the phase problem in the crystallographic analysis of FEA1 crystals. This study will lead to further structural studies of FEA1 to understand its function and its links to the iron-assimilation pathway.

## Figures and Tables

**Table 1:** Information on the recombinant expression of FEA1

Source organism	*C. reinhardtii*
DNA source	DNA fragment generated by synthesis
Expression vector	pASK-IBA2
Expression host	*E. coli* strain Rosetta2 (DE3)
Complete amino-acid sequence of the construct produced†	QPTTTGTRFEGFSYAGNVIGYVNMTMDYCDIKAAMAAGNFTEALSIYSTGKNSFSGLARRTFFRFASYITANGSVEPLHDSILAGKDTSSLDAAIRAALADGKATLAAGLQVGTLKYHLHEVDEAYNKIKTYLADGTGNLTNLVSDASGAPHNVDEAWALWAGGAANNCGTLSGWASSLGAAMGTTFLGKSYVNTAMINTVNEMLAAARLSTLNIQAYDAARTNEVRLLTLLGLQGVSVAAYTADAAAACKRPAAEVEDAKTMIAVHWAYLEPMLKLRNFKASAVTELHHQLTASKLSYKKVAAAVKGVLSAMGRRSSELGAPQSAIIAANWKCSSKTLRSIALEVDLQGDHGLSAWSHPQFEK
UniProt identifier	Q9LD42

† The 21-residue Strep-tag added to the C-terminus of the native sequence is underlined.

**Table 2:** Conditions for crystallization of FEA1

Method	Hanging-drop vapor diffusion
Plate type	VDX48 plate with sealant (Hampton Research)
Temperature (K)	277
Protein concentration (mg ml^−1^)	10
Buffer composition of protein solution	40 m*M* Tris–HCl pH 7.5
Composition of reservoir solution	0.2 *M* ammonium sulfate, 0.1 *M* imidazole–HCl pH 6.5, 11%(*w*/*v*) PEG 3350, 30%(*v*/*v*) MPD
Volume and ratio of drop	2 µl, 1:1 ratio of protein:reservoir solution
Volume of reservoir (µl)	150

**Table 3:** Data collection and processing Values in parentheses are for the outer shell.

	Native	S-SAD
Diffraction source	BL44XU, SPring-8	BL-1A, PF
Wavelength (Å)	0.90000	2.70000
Temperature (K)	100	100
Detector	EIGER X 16M	EIGER X 4M
Crystal-to-detector distance (mm)	200	61.5
Rotation range per image (°)	0.1	0.1
Exposure time per image (s)	0.1	0.01
Resolution range (Å)	38.30–1.90 (2.01–1.90)	49.04–2.69 (2.76–2.69)
Space group	*C*2	*C*2
*a*, *b*, *c* (Å)	85.75, 155.94, 129.53	85.44, 155.32, 129.22
α, β, γ (°)	90, 102.28, 90	90, 101.84, 90
Total no. of reflections	381316 (61723)	3774689 (40196)
No. of unique reflections	129679 (20765)	88102 (5191)
Multiplicity	2.9 (2.9)	42.8 (7.7)
Completeness (%)	98.9 (98.5)	97.4 (78.3)
Mean *I*/σ(*I*)	12.0 (1.4)	24.4 (1.8)
*R*_merge_	0.06 (0.91)	0.15 (0.92)
*R*_meas_	0.07 (1.11)	0.15 (0.98)
CC_1/2_ (%)	99.8 (55.0)	99.9 (63.8)
